# The perspectives of clinical staff and bereaved informal care-givers on the use of continuous sedation until death for cancer patients: The study protocol of the UNBIASED study

**DOI:** 10.1186/1472-684X-10-5

**Published:** 2011-03-04

**Authors:** Jane Seymour, Judith Rietjens, Jayne Brown, Agnes van der Heide, Sigrid Sterckx, Luc Deliens

**Affiliations:** 1 School of Nursing, Midwifery and Physiotherapy, University of Nottingham, Queen's Medical Centre, Derby Road, Nottingham, NG7 2UH, UK; 2 End-of-Life Care Research Group, Ghent University and Vrije Universiteit Brussel, Laarbeeklaan 103, 1090 Jette, Belgium; 3 Department of Public Health, Erasmus MC, University Medical Centre, P.O. Box 2040, 3000 CA, Rotterdam, Netherlands; 4 Ghent University, Bioethics Institute Ghent, Blandijnberg 2, 9000 Gent, Belgium

## Abstract

**Background:**

A significant minority of dying people experience refractory symptoms or extreme distress unresponsive to conventional therapies. In such circumstances, sedation may be used to decrease or remove consciousness until death occurs. This practice is described in a variety of ways, including: 'palliative sedation', 'terminal sedation', 'continuous deep sedation until death', 'proportionate sedation' or 'palliative sedation to unconsciousness'. Surveys show large unexplained variation in incidence of sedation at the end of life across countries and care settings and there are ethical concerns about the use, intentions, risks and significance of the practice in palliative care. There are also questions about how to explain international variation in the use of the practice. This protocol relates to the UNBIASED study (UK Netherlands Belgium International Sedation Study), which comprises three linked studies with separate funding sources in the UK, Belgium and the Netherlands. The aims of the study are to explore decision-making surrounding the application of continuous sedation until death in contemporary clinical practice, and to understand the experiences of clinical staff and decedents' informal care-givers of the use of continuous sedation until death and their perceptions of its contribution to the dying process. The UNBIASED study is part of the European Association for Palliative Care Research Network.

**Methods/Design:**

To realize the study aims, a two-phase study has been designed. The study settings include: the domestic home, hospital and expert palliative care sites. Phase 1 consists of: a) focus groups with health care staff and bereaved informal care-givers; and b) a preliminary case notes review to study the range of sedation therapy provided at the end of life to cancer patients who died within a 12 week period. Phase 2 employs qualitative methods to develop 30 patient-centred case studies in each country. These involve interviews with staff and informal care-givers closely involved in the care of cancer patients who received continuous sedation until death.

**Discussion:**

To our knowledge, this is one of the few studies which seek to take a qualitative perspective on clinical decision making surrounding the use of continuous sedation until death and the only one which includes the perspectives of nurses, physicians, as well as bereaved informal care-givers. It has several potential strengths, weaknesses, opportunities and threats associated with the specific design of the study, as well as with the sensitive nature of the topic and the different frameworks for ethical review in the participating countries.

## Background

A significant minority (cancer research indicates between one tenth and one quarter) of dying people experience refractory symptoms (such as agitated delirium) or extreme distress which is unresponsive to conventional therapies [1,2]. In such circumstances, sedation may be used to decrease or remove consciousness. Internationally, prevalence studies to ascertain the percentage of patients receiving sedation for refractory symptoms at the end of life report variation in use of between 15% to 60% [3-5]. The wide variation in reported prevalence is likely to be due in part to differences in the care setting, patient populations, methods and the definitions applied. These definitions include: 'palliative sedation', 'terminal sedation', 'continuous deep sedation until death', 'proportionate sedation' and 'palliative sedation to unconsciousness' [6].

One group of studies exists that have enabled comparisons to be made; these focus on quantifying types of medical end of life decisions, using questionnaires to ask physicians to describe the decision-making and care for recent deaths they attended [7-9]. One of these studies, looking at representative samples of death certificates in six countries in Europe (excluding the UK), employed a narrow definition of 'continuous and deep sedation until death' to compare physicians' reports, and showed a relatively narrow band of variation in prevalence of between 2.5% to 8.5% of deaths [8]. A survey in the UK employing the same definition found that 16.5% of the 2,923 respondents reported use of continuous deep sedation until death, although study limitations may have led to over estimation of prevalence [7].

An important focus of debate surrounding the practice concerns the relationship between continuous sedation until death for refractory symptoms or distress and any adverse consequences which may result from its use (known as 'double effect' issues) [10]. These include the risk of paradoxical agitation, the loss of ability of the patient to interact and the hastening of an expected death [10-12]. While a systematic review of sedative use [13] and comparative studies [4,14] have shown no relationship to exist between the use of sedation for refractory symptoms or distress and timing of death, in the Netherlands and Belgium there is evidence to suggest that continuous sedation until death may sometimes be used by clinicians both as an alternative for euthanasia and to hasten an expected death [15,16]. More broadly, the ethical dimensions of the therapy are increasingly being debated [17-19].

Ethical concerns surrounding the practice have resulted in the publication of a number of guidelines or frameworks for clinical practice [10,20,21]. These recommend that the use of continuous sedation until death for refractory symptoms or extreme distress should occur only when the patient's disease is irreversible and advanced, that benzodiazepines should be the drug of first choice, that decisions to use artificial hydration should be considered separately and offered to sedated patients only when the benefits outweigh harm, and that physicians with little expertise in sedation seek advice from palliative care specialists before initiating continuous sedation until death.

There have been calls for research to examine clinical practice in a qualitative manner to complement the large scale survey studies described above and provide explanatory data about the intentions, practices and understandings associated with continuous sedation until death and its perceived contribution to the management of refractory symptoms and distress [22]. There is, with a few exceptions, little research exploring the understandings, intentions and beliefs of physicians and nurses regarding the use of continuous sedation until death or the perceptions and experiences of bereaved informal care-givers.

Those studies which do exist of health care staff suggest that clinicians, whether physicians or nurses, struggle with the meaning and significance of sedation use in end of life care [23], are concerned about its possible link to practices which may end life prematurely [24], or have objections to the practice on the grounds of religious belief or moral conviction [25] or because of ideas about appropriateness of the practice for certain 'types' of suffering [26,27]. There is evidence that physicians and nurses have different experiences of the decision-making process relating to continuous sedation until death [28] and of international differences in clinicians' understandings and perceptions [29]. One small scale qualitative interview study of clinicians and researchers in end-of-life care in England, Belgium and the Netherlands [30] found that beliefs and attitudes regarding the practice were affected by the socio-legal context of end-of-life care practice within each country and that judgements about refractoriness were regarded as likely to be affected by resource availability.

Studies of bereaved informal care-givers and members of the general public [31,32] indicate that it is preferable to provide information in advance about the need to sedate, its contribution to the care of the patient and its consequences. One study in Japan shows that one quarter of families who witnessed the use of sedation for refractory symptoms experienced significant distress [33].

This protocol relates to the UNBIASED study (**U**K **N**etherlands **B**elgium **I**ntern**a**tional **Sed**ation Study), which comprises three linked studies with separate funding sources in the UK, Belgium and the Netherlands designed to explore these issues. The UNBIASED study is part of the European Association for Palliative Care Research Network [34]. The study design has been scientifically peer reviewed as part of the grant application process by the Economic and Social Research Council (UK); Fund for Scientific Research (Belgium), the Netherlands Organisation for Scientific Research and the Netherlands Organisation for Health Research and Development.

### Aims of the study

• To explore decision-making surrounding the application of continuous sedation until death in contemporary clinical practice.

• To understand the experiences of clinical staff and decedents' informal care-givers of the use of continuous sedation until death and their perceptions of its contribution to the dying process.

### Research Questions

For cancer patients at home, in hospitals and in hospice settings:

1. In accounts of clinical practice, what do physicians and nurses mean by sedation in end of life care?

2. What are the clinical characteristics of the application of continuous sedation until death according to physicians and nurses?

3. How do bereaved informal care-givers, physicians and nurses describe decedents' care at the end of life and the contribution made by continuous sedation until death?

4. Who are, according to physicians, nurses and bereaved informal care-givers, the key people involved in the decision-making with regard to the use of continuous sedation until death, and what role do they play?

5. What accounts do physicians and nurses give about communication and consent in relation to the use of continuous sedation until death?

6. What reasons are reported for the use of continuous sedation until death by physicians, nurses, and bereaved informal care-givers, and what reasons for the use of continuous sedation until death are described in clinical records and in clinical guidelines and protocols?

7. What moral and legal issues emerge from the accounts of physicians, nurses and bereaved informal care-givers about decisions taken regarding continuous sedation until death?

8. What aspects of context, attitudes and experience facilitate or constrain decision-making according to the accounts of physicians, nurses and bereaved informal care-givers?

9. What are the differences between Belgium, the Netherland and the United Kingdom with respect to the findings of research questions 1 to 8?

## Methods/design

### Study population

There are four categories within the study population: 1) deceased adult cancer patients; 2) informal care-givers of deceased patients; 3) nurses involved; and 4) physicians Involved in care-giving.

### Study settings

The study settings include hospitals, expert palliative care units and the domestic home.

### Design

The UNBIASED study has two phases: an exploratory phase (1) and a case study phase (2).

### Phase 1

Phase 1 of the study has been designed to set the patient-centred case studies conducted in phase 2 in a broader context of understanding. It includes a series of focus groups and case notes review.

### Focus groups

In the focus groups, attention is directed at gaining an understanding of informal care-givers' and health care professionals' beliefs, perceptions, and understandings of sedation in end-of-life care for adult cancer patients; and, in the case of health care professionals, also at gaining an appreciation of their experiences of continuous sedation, and views of the factors that facilitate or constrain their involvement in its use. Depending on the context in each participating country, and what data already exist from earlier work, this has been achieved by the following means:

a) Focus groups with health care professionals in a variety of care settings. The UNBIASED study involves six focus groups with physicians and nurses in the UK and four in Belgium.

b) Focus groups with informal care-givers. Three focus groups with informal care-givers have been undertaken in the Netherlands as part of the UNBIASED collaboration.

### Case notes review

The exploratory phase also includes review in all three countries of the case notes of adults who have died from cancer during a given period of 12 weeks in each of the study settings, i.e. acute hospital care, expert palliative care and domestic home settings. The purpose of the case notes review is to gain an understanding of the prevalence and types of and reasons for sedation therapy in the end-of-life care of cancer patients.

### Phase 2

Phase 2 of the study in all three countries takes the form of in-depth retrospective case studies of 30 adults who died from cancer (10 from each type of study setting), where sedation was administered continuously for otherwise refractory symptoms or distress and was in place at the time of their death. Case studies have been found to shed detailed light on problems in palliative and end-of-life research [35]. They are suitable for exploring practically and ethically complex situations involving a variety of perspectives. Detailed insights from well-constructed case studies also have an explanatory potential [36].

Each patient centred case study entails three elements: a) the reasons for the use of continuous sedation, the drugs used, and notes about the content of decision-making, as recorded in the medical notes; b) interviews with key physicians and nurses involved in the decedent's care to examine their perspectives on the end of life management and their role in decision-making; and c) interviews with bereaved informal care-givers to explore their recollection and experiences of the decedent's end of life care.

### Inclusion criteria and sample sizes

#### Phase 1 focus group participants

Nurses and physicians working in hospitals, expert palliative care units or in a community setting, who have experience of giving care to cancer patients receiving sedation in the last days of life, and informal care-givers whose companion received continuous sedation prior to their death and who died less than one year ago. Care will be taken to minimise overlap between participation in the focus groups and the patient-centred case studies in phase 2 (see below). Sample sizes provided here are indicative. Sample sizes for nurses and physicians taking part in focus groups are likely to be up to 30 in each group. Up to 20 informal care givers are included in focus groups.

#### Phase 1 case notes review

The medical and nursing records of all people with a primary diagnosis of cancer over 18 years old, who died within a designated 12 week period and were cared for in participating hospitals, expert palliative care units or at home. The numbers of case records included in the search will be dependent upon the number of cancer deaths occurring within the designated 12 week period.

#### Phase 2 patient centred case studies

##### The deceased patients

Thirty patients with a primary diagnosis of cancer over 18 years old, who died in participating hospitals, expert palliative care units or at home (10 patients in each type of setting) and for whom sedation with the intention to decrease awareness was administered continuously with sedating medications (benzodiazepines or propofol, but not morphine) to alleviate otherwise uncontrollable symptoms (either physical or psychological/existential), and for whom the sedation was in place at the time of death.

##### Nurses and Physicians

Physicians (n = 30) and nurses (n = 30) who took a key role in the care of the person who died are invited for interview, within six weeks of the death. Where more than one clinician was closely involved, additional interviews may be conducted.

##### Informal care-givers

Individuals (n = 30), over the age of 18 years who are not professional care-givers but by default or choice were involved in the care and/or emotional support of the person who died, and who belong to that person's immediate social and familial network [37], are invited to take part in the study at least 3 months following the death.

### Study procedure: Phase 1

#### Case notes review

The case notes review covers a designated 12 week period. All cancer deaths occurring during this period are identified by clinical coding used in medical records departments. Documentary evidence about symptoms, types and duration of treatment provided and evidence of end of life decisions during the last week of life are extracted on a proforma, with particular attention paid to the use of sedation therapy (see additional file 1).

### Focus groups with nurses, physicians and informal care-givers

Focus groups are held with physicians and nurses who have experience of working with cancer patients who have received continuous sedation until death. Similarly, focus groups are held with informal care-givers whose relative or friend received continuous sedation until death in the past year. Discussions take approximately one hour and are audio recorded and transcribed in full. Additional files 2 and 3 indicate the areas of discussion for the focus groups.

### Study procedure: Phase 2

#### Case identification

On a weekly basis, in close collaboration with the research teams, and using a purposive sampling approach [38], potential cases of deceased patients who received continuous sedation for otherwise uncontrollable symptoms are identified by senior clinical staff working in the three study sites. This assists in the identification of a range of cases of interest, where sedation was both planned and unplanned, and with variation in depth and length of sedative therapy.

### Interviews with key physicians, nurses and informal carers

Invitations are extended to the physicians and nurses most involved in the care of the deceased patients as soon as possible, i.e. within six weeks, to maximize recall. Physicians and nurses are invited to use the patient records to support them in their recollection of the case and to provide other relevant information to the researchers about the case in an anonymous manner. The interviews focus on the nurses' and physicians' recollections of the care of the decedent, the interactions with their informal care-givers immediately before death, and the decision-making process relating to the use of continuous sedation (see additional file 4). We seek to uncover the difficulties and problems facing clinicians in the practice of continuous sedation until death and how they sought to resolve them, as well as the environmental barriers to or facilitators of decisions made and wider perceptions of death, dying and bereavement.

At least 3 months after the death of the patient, the person identified as their closest informal care-giver is invited to take part in the study via a letter sent on behalf of the research team by the appropriate senior clinician. This invitation explains the aims of the research and sets out the areas covered in the interview. Individuals are asked to contact the research team if they are interested in taking part. Telephone contact is then made to answer questions, explain what involvement in the study will involve and arrange a time and place for an interview, where this is agreed. Additional file 5 outlines the interview guide for use in interviews with bereaved family care-givers, which has been carefully designed in recognition of the fact that some care-givers may not recall the use of sedation therapy.

For all interviews, written informed consent will be obtained.

### Data analysis

Data from the case notes (in the phase 1 case notes review and phase 2 case studies) is entered into SPSS™ for basic descriptive analysis. With consent from the participants, the interviews and focus groups are audio recorded and subsequently transcribed. Transcripts are analysed with the aid of the qualitative data analysis package NVIVO™ and using Strauss and Corbin's [39] constant comparative method to generate codes, categories and themes. This involves close collaboration between the national teams. In relation to the phase 2 case studies, each set of related interviews and case note reviews will be compiled into a case study. Each transcript will be initially analysed by the researcher who undertook the interview. Emerging categories and themes will be subsequently verified by another member of the research team, and then on the basis of consultation internationally, to enhance validity of the data. Encrypted anonymised findings from the UK, Belgium and the Netherlands will be shared between the researchers undertaking this study in parallel in order to gain a wider international perspective and to subject the data to an ethical analysis (see next section).

Key themes derived from the cross case analysis and the international analysis will be presented to clinical staff from the research sites included in the study and to audiences of key stakeholders to develop recommendations for practice.

### Ethical aspects of the study and ethical analysis of the data

This study requires that particular sensitivity be shown when undertaking the interviews. Confidentiality are of the utmost importance, given the sensitive nature of this subject. In interviewing bereaved people, we will ensure that each person is provided with details of local bereavement support resources. The research interviewer, with the interviewee's permission, will telephone them three days after the interview both to check well-being and to answer any questions which the interview may have raised. Where the interviewee wishes, contact will be made with the clinical teams who provided care to the decedent to help address such questions. Support and supervision will be provided to the interviewers, since such fieldwork can cause stress to the research team. Research ethics committee approval (using the systems in place in each country) has been sought. In addition, in the UK it has been necessary to gain 'research governance' approval from the organisations within which each study site is located.

As noted earlier, the data generated by the focus group discussions as well as the interviews will be subjected to an ethical analysis by the ethicists in the team. The purpose of this analysis is to provide instruments that can be used to judge the validity of moral arguments in favour of or against continuous sedation at the end of life, as well as to distinguish between various medical practices at the end of life on the basis of morally relevant criteria (e.g. request by the patient, life-shortening effect, etc.).

This analysis will inform a broader ethical study of sedation which is being carried out in parallel with the empirical study described in this protocol. The overarching research question of the ethical study is: under which conditions is continuous sedation until death ethically justified? More specifically, based on the empirical data, the situations in which continuous sedation until death is considered ethically acceptable by medical practitioners and on what grounds, will be investigated. The justifications offered will be analysed with regard to their internal consistency as well as the respects in which they differ from ethical justifications of other medical practices at the end of life.

The various situations, in which continuous sedation at the end of life is used, will be studied with a focus on the following criteria:

*-Patient autonomy and informed consent*:

• Does continuous sedation at the end of life require informed consent if the patient is competent?

• What about decision making where the patient is incompetent? Which side constraints should apply regarding surrogate decision-making?

• What role could advance care planning play in this context?

-*Proportionality of the sedation*:

• How should the traditional requirement of proportionality of sedation to the patient's symptoms be interpreted and what are its justifications in different situations?

- *The doctrine of double effect and the doctrine of acts versus omissions*:

• To what extent, and under what conditions, does the practice of continuous sedation until death 'fit' with these doctrines;

• Are only intentions relevant in this regard?

• What is the importance of (the lack of) intentions in cases where the sedation foreseeably results in shortening the life of the patient?

- *Compatibility with legal provisions*:

• There seems to be widespread agreement that continuous sedation until death is part of "normal medical practice" (like for example, non-treatment decisions and unlike, for example, euthanasia), but what about the legal status of cases in which sedation may be used as an alternative to euthanasia or to life-ending without request?

• Which normative rules ought to apply here?

## Discussion

The objectives of the international UNBIASED study are to explore decision-making surrounding the application of continuous sedation until death in contemporary clinical practice, and to understand the experiences of clinical staff and decedents' informal care-givers of the use of continuous sedation until death and their perceptions of its contribution to the management of dying. To realize these objectives, a two-phase qualitative study has been designed including focus groups and in-depth interviews; in addition some quantitative data is gathered from clinical records by way of a preliminary case note review to understand the range of sedation therapy provided at the end of life to cancer patients who died within a 12 week period. The first results of the study are expected in 2011.

This study has several potential strengths, limitations, opportunities and threats associated with the specific design of the study, as well as with the sensitive nature of the topic and these are outlined below.

### Strengths

To our knowledge, this is one of the few studies which seek to take a qualitative perspective on clinical decision-making surrounding the use of continuous sedation until death and the only one which includes the perspectives of nurses, physicians, and bereaved informal care-givers. Research using qualitative methodologies to provide understanding about the nuances and challenges of end of life decision making has featured comparatively rarely in the medical research literature, although it has a long tradition in the field of the sociology of health and illness [40] and, to a lesser but increasing degree, in the study of bioethics [41]. Our international collaboration brings together a team of ethicists, sociologists, psychologists, public health researchers and clinicians (both physicians and nurses) to lend not only our disciplinary perspectives to this difficult subject but also to combine what we have learnt from the application of different methodological techniques in palliative and end-of-life care research. In addition, we contribute these from differing national and cultural contexts, adding a further dimension to the study which we anticipate will enrich its outcomes.

### Limitations

There are a number of limitations to this study, which stem partly from the sensitivity of the subject and its relatively small scope, and partly from the methodological challenges of studying a phenomenon which is associated with such intense debate about its definition, indications and practice. It is likely that we will have incomplete data in terms of gathering perspectives from both professionals and informal care-givers. Moreover, because this is a qualitative study based on relatively small numbers of cases, we will not be able to generalise in a statistical sense from our findings; instead our focus is primarily on providing contextualised insights on the basis of case to case transfer and analytical or theoretical grounds [42]. Perhaps the most challenging and yet the most interesting aspect concerns matters of definition and meaning: achieving consensus and thus comparability across cases and between national studies is likely to be difficult given the range of understandings 'out there' about the meaning and appropriateness of sedation in end-of-life care and the different interpretations of refractory symptoms and distress [18].

### Opportunities

Completion of this project will enable a clinically, ethically and sociologically informed understanding of some important but little studied contemporary practices associated with end-of-life care and contribute to efforts to develop research methodologies which can capture the ethical and clinical issues involved in end-of-life care decision-making at the bedside. We hope that our findings will inform debate and the development of practice and policy in end-of-life care via collaboration with the European Association of Palliative Care and other key national and international stakeholders. The establishment of the UNBIASED research consortium provides an important opportunity to conduct an international study of a contentious topic and provides a potential framework for future collaborations with which research teams from other counties may wish to engage via duplication of (parts of) the study.

### Threats

The threats to the successful conduct of this study relate mainly to the differing ethical review frameworks and procedures encountered in each country. We have had to make significant adjustments to the study design (especially in terms of ways of accessing deceased patients' clinical records) to comply with the strict yet somewhat different demands imposed by ethical and governance review committees in the three national contexts. These procedures specifically apply to researchers not employed as members of the clinical teams responsible for providing care to patients included in the study. This risks imposing additional costs on each national project and has created a delay in terms of commencement of fieldwork; fortunately this has been a similar experience in each country. They also introduce a possibility of bias, because the myriad of individual requirements imposed by different institutional review bodies. Considerable and continuing efforts are therefore necessary to ensure comparability of the national studies, as the study teams seek to comply with local and national requirements in the conduct of their projects.

We hope that others will be able to use this protocol to replicate the study, with necessary local adaptations, to enable further comparisons.

The international debate on medical decision-making at the end of life has caused substantial and profound differences of opinion between opinion leaders, different professions, the public and different countries. There are now emerging efforts to facilitate dialogue in spite of these differences about how best to collaborate and communicate in reaching the common goal of better end-of-life care across Europe. This project will contribute to this by examining and comparing the issues thrown up by different models and approaches to end of life care, and reporting these to stakeholders engaged in developing proposals for policy and practice in this field. This study is one example of the emergent opportunities for partnerships between colleagues across a wide span of disciplines to further the critical and informed debate about a question that concerns us all: how to shape the compassionate care of persons in the final stages of life and their companions.

Please contact the lead author for more information about this study and to discuss any interest in joining this collaboration.

## Competing interests

The authors declare that they have no competing interests.

## Authors' contributions

The authors, who are study Steering Group representatives, are drawn from three national research teams which jointly designed the study, based on an original outline study proposal submitted for funding in the UK by JS. JS, JR and JB co-wrote the first draft of the paper. SS wrote the section on ethical analysis of the data. AH, SS and LD edited the paper and agreed its final content. All authors read and agreed the final content of the manuscript.

## Pre-publication history

The pre-publication history for this paper can be accessed here:

http://www.biomedcentral.com/1472-684X/10/5/prepub

## Supplementary Material

Additional file 1 **Box A: Key headings for the case notes review of adults who died of cancer**. Click here for file

Additional file 2 **Box B: Aide memoire for focus group with physicians and nurses who have experience of sedation therapy**. Click here for file

Additional file 3 **Box C: Aide memoire for focus group with relatives whose relatives experienced sedation therapy**. Click here for file

Additional file 4 **Box D: Aide memoire interviews with physicians and nurses, questions (bold) and subsidiary prompts**. Click here for file

Additional file 5 **Box E: Aide memoire interviews with informal care-givers, questions (bold) and subsidiary prompts**. Click here for file
