# Supplementary material for: The perspectives of clinical staff and bereaved informal care-givers on the use of continuous sedation until death for cancer patients: The study protocol of the UNBIASED study
Source: BMC Palliat Care. 2011 Mar 4;10:5. doi: 10.1186/1472-684X-10-5 (PMC3056823; doi:10.1186/1472-684X-10-5)
Supplement: Additional file 1 — Box A: Key headings for the case notes review of adults who died of cancer. [file 1472-684X-10-5-S1.DOC]

**Box A: Key headings for the case notes review of adults who died of cancer**

**Demographic Information**

- Gender
- Age at death
- Ethnicity
- Place of death (e.g. home, hospital)
- Mode of death (e.g. sudden, expected)
- Home circumstances (e.g. lived alone, with spouse)

**The way in which the death of the patient is described**

- E.g. relevant comments, final primary diagnosis

**Clinical Information relating to the last week of life**

- Evidence of ‘Do not resuscitate’ order
- Symptoms (description, duration, intensity, action taken)
- Therapeutic interventions (e.g. hydration, nutrition)

**Medication (both as prescribed and as administered)**

- Sedation (date commenced and finished, reasons for commencing / stopping)
- Effects of sedation (level of sedation, other effects)
- Other medication (date commenced and finished , reasons for commencing/ stopping)

**Decision Making**

- Record of discussion of sedation and other related issues between health care professionals and next of kin
- Use of clinical guidelines (e.g. Liverpool Care Pathway; EAPC framework; local policies)
