# Supplementary material for: The perspectives of clinical staff and bereaved informal care-givers on the use of continuous sedation until death for cancer patients: The study protocol of the UNBIASED study
Source: BMC Palliat Care. 2011 Mar 4;10:5. doi: 10.1186/1472-684X-10-5 (PMC3056823; doi:10.1186/1472-684X-10-5)
Supplement: Additional file 2 — Box B: Aide memoire for focus group with physicians and nurses who have experience of sedation therapy. [file 1472-684X-10-5-S2.DOC]

**Box B: Aide memoire for focus group with physicians and nurses who have experience of sedation therapy**

**Types of Sedation**

*Which types of sedation do you use in end of life care?*

*How do you define these?*

**Discussion of hypothetical cases^1^ of continuous sedation until death**

- *Deep versus lighter continuous sedation until death*
- *Longer life expectancy versus shorter life expectancy,*

*How would you define what the doctor did in this case?*

*Would you act in a similar manner if you cared for a similar patient? Why / why not?*

**Own experiences of continuous sedation until death**

*How are you involved in the use of continuous sedation until death in end-of-life care?*

*For which types of patients and in which situations?*

*Are there any aspects that facilitate or constrain your involvement in the use of continuous sedation until death?*

**General Discussion and Close**

1. *Available from the authors on request*
