# Supplementary material for: The perspectives of clinical staff and bereaved informal care-givers on the use of continuous sedation until death for cancer patients: The study protocol of the UNBIASED study
Source: BMC Palliat Care. 2011 Mar 4;10:5. doi: 10.1186/1472-684X-10-5 (PMC3056823; doi:10.1186/1472-684X-10-5)
Supplement: Additional file 3 — Box C: Aide memoire for focus group with relatives whose relatives experienced sedation therapy. [file 1472-684X-10-5-S3.DOC]

**Box C: Aide memoire for focus group with relatives whose relatives experienced sedation therapy**

**Decision-making**

*How was the decision to use continuous sedation until death for your relative or friend made?*

*(How) were you informed about the use of sedation?*

**Performance of sedation**

*Can you describe the performance of the sedation? (depth, duration etc)*

*How were you yourself involved in the use of sedation? (taking care, monitoring etc..)*

**Evaluation**

*When you look back at the death of your loved one, how did the use of sedation contribute?*

*How did you yourself experience the use of sedation?*

**General discussion and close**
