# Supplementary material for: The perspectives of clinical staff and bereaved informal care-givers on the use of continuous sedation until death for cancer patients: The study protocol of the UNBIASED study
Source: BMC Palliat Care. 2011 Mar 4;10:5. doi: 10.1186/1472-684X-10-5 (PMC3056823; doi:10.1186/1472-684X-10-5)
Supplement: Additional file 4 — Box D: Aide memoire interviews with physicians and nurses, questions (bold) and subsidiary prompts. [file 1472-684X-10-5-S4.DOC]

**Box D: Aide memoire interviews with physicians and nurses, questions (bold) and subsidiary prompts**

| **I would like to talk to you about patient x who died (time ago). Could you tell me what you remember about the care of patient X?** *What symptoms and problems did they have? How were these managed? How were you involved?*  **We understand that they received sedation (give specifics from the notes). Could you tell me what you recall about how the decision to use continuous sedation was made?** *Why was sedation used?*  *Apart from the patient’s condition, where there any other factors that influenced the decision to start sedation? Who made the decision?*  *Who was involved (physicians, nurses, patient, family, others) and what role (s) did they play? Is that the usual role they play in decisions in sedation therapy? How did their role and yours interact? Can you recall any alternative treatment options to sedative therapy being considered?*  **What do you recall about the use of sedation for patient x?**  *How effective were the drugs in relieving symptoms? When the sedative therapy was started, how long was it intended to continue? How did that differ from what actually happened? Can you recall how deeply or lightly patient X was sedated? Can you recall whether the use of sedation was linked to any other decisions about end of life care? (e.g. artificial nutrition and hydration use/ non use)*  **When you look back on the quality of care of patient X, what do you think was the contribution of sedation?**  *How effective were the drugs in relieving symptoms? What was the perceived contribution of sedation to the death?*  **Do you think that patient X was a typical or atypical example of a patient for whom sedation was necessary at the end of life?**  *Why is that?*  **If you were involved in the care of this patient again would you do anything differently, or like to see anything done differently?**  *Why is that?*  **When you think of the term ‘sedation in end of life care’ for cancer patients, what comes to mind for you?**  **Can you tell me about your general experiences of the use of sedation in end of life care for patients dying of cancer in this (hospital, hospice, practice/ team)?**  *Is sedation over/ under used? How effective is sedation? Have you personally have ever found the use of sedation therapy to be difficult? In your opinion are there any moral issues involved in providing sedation?*  **Is there anything else that you think is important that we haven’t covered or that you expected me to ask you about?** |
| --- |
